# Using administrative health data to describe colorectal and lung cancer care in New South Wales, Australia: a validation study

**DOI:** 10.1186/1472-6963-12-387

**Published:** 2012-11-09

**Authors:** David E Goldsbury, Katie Armstrong, Leonardo Simonella, Bruce K Armstrong, Dianne L O’Connell

**Affiliations:** 1 Cancer Research Division, Cancer Council NSW, PO Box 572, Kings Cross, NSW 1340, Australia; 2 Sydney Medical School, The University of Sydney, Sydney, NSW 2006, Australia; 3 School of Public Health and Community Medicine, Faculty of Medicine University of New South Wales, New South Wales, NSW 2052, Australia; 4 School of Medicine and Public Health, Faculty of Health University of Newcastle, New South Wales, NSW 2308, Australia

**Keywords:** Linked data, Validation, Colorectal cancer, Lung cancer, Investigative procedures, Disease stage, Surgery, Chemotherapy, Radiotherapy, Comorbidities

## Abstract

**Background:**

Monitoring treatment patterns is crucial to improving cancer patient care. Our aim was to determine the accuracy of linked routinely collected administrative health data for monitoring colorectal and lung cancer care in New South Wales (NSW), Australia.

**Methods:**

Colorectal and lung cancer cases diagnosed in NSW between 2000 and 2002 were identified from the NSW Central Cancer Registry (CCR) and linked to their hospital discharge records in the NSW Admitted Patient Data Collection (APDC). These records were then linked to data from two relevant population-based patterns of care surveys. The main outcome measures were the sensitivity and specificity of data from the CCR and APDC for disease staging, investigative procedures, curative surgery, chemotherapy, radiotherapy, and selected comorbidities.

**Results:**

Data for 2917 colorectal and 1580 lung cancer cases were analysed. Unknown disease stage was more common for lung cancer in the administrative data (18%) than in the survey (2%). Colonoscopies were captured reasonably accurately in the administrative data compared with the surveys (82% and 79% respectively; 91% sensitivity, 53% specificity) but all other colorectal or lung cancer diagnostic procedures were under-enumerated. Ninety-one percent of colorectal cancer cases had potentially curative surgery recorded in the administrative data compared to 95% in the survey (96% sensitivity, 92% specificity), with similar accuracy for lung cancer (16% and 17%; 92% sensitivity, 99% specificity). Chemotherapy (~40% sensitivity) and radiotherapy (sensitivity≤30%) were vastly under-enumerated in the administrative data. The only comorbidity that was recorded reasonably accurately in the administrative data was diabetes.

**Conclusions:**

Linked routinely collected administrative health data provided reasonably accurate information on potentially curative surgical treatment, colonoscopies and comorbidities such as diabetes. Other diagnostic procedures, comorbidities, chemotherapy and radiotherapy were not well enumerated in the administrative data. Other sources of data will be required to comprehensively monitor the primary management of cancer patients.

## Background

Colorectal and lung cancers are the second and fifth most common cancers in New South Wales (NSW), Australia’s most populous state. In 2008, the two cancers together accounted for 22% of all new cancers and 33% of cancer deaths [1]. Monitoring treatment patterns and evaluating associated outcomes is a necessary requirement for improving care amongst these cancer patients. While population-based patterns of care surveys are valuable for this purpose, they are resource-intensive and provide only a snapshot of care. The use of linked routinely collected administrative health data, if sufficiently reliable, would be more efficient, potentially cost-effective and allow for the monitoring of cancer care over time.

The NSW Central Cancer Registry (CCR) and NSW Admitted Patient Data Collection (APDC) are two routinely collected administrative data sources that together could provide information on cancer treatment in NSW. A recent validation study found these data sources accurately recorded radical prostatectomy and brachytherapy treatment for prostate cancer patients, but not external beam radiotherapy [2]. An earlier breast cancer study described reasonable enumeration of surgery for breast cancer [3]. However there is little other published material investigating the validity of these data sources for describing patterns of cancer care, despite their increasing use for this purpose e.g [4-6].

Cancer stage information is vital for assessing the appropriateness of care. The previous prostate cancer study found a high proportion of tumours had unknown stage in the NSW Cancer Registry [2]. Another study reported 70% agreement between the CCR and colorectal cancer stage collected in a survey of treating clinicians [7]. Similar studies in another Australian state and New Zealand reported around 80% agreement/accuracy of the pathology-based colorectal cancer staging information that is reported in cancer registries, suggesting it is a valid source of high-level stage information [8,9].

Here we report on the validity of the administrative data for recording diagnostic procedures and treatment received by colorectal and lung cancer patients, along with cancer stage and selected comorbidities for lung cancer patients. This study adds to the limited existing literature regarding the use of these data to assess and monitor patterns of cancer care over time. Given the potential utility of these population-based data sources for this purpose, with only a fraction of the resources required by other methods, this study makes an important contribution to the literature.

## Methods

### Patterns of care study data

Two population-based studies carried out by Cancer Council NSW collected detailed treatment data for colorectal and lung cancer patients diagnosed in NSW. The NSW Colorectal Cancer Care Survey (called the “colorectal cancer survey”) collected data on the patterns of care for colorectal cancer patients notified to the CCR between February 2000 and January 2001 [10]. The NSW Lung Cancer Patterns of Care study (called the “lung cancer survey”) collected treatment data for lung cancer cases from the CCR diagnosed between November 2001 and December 2002 [11].

For both studies, clinicians who treated these patients were identified from CCR notifications. The physicians were then sent questionnaires seeking information on the patient’s initial presentation, investigations and surgery, chemotherapy and radiotherapy in the primary treatment phase. A field officer collected this information from clinicians’ records where necessary and feasible. Patients normally resident outside NSW were excluded. In the colorectal cancer survey, treating institutions were identified and categorised by type and location. In the lung cancer survey, the comorbidities recorded were conditions assessed at initial presentation that were likely to impact on the patient’s disease or treatment; the patient’s performance status and weight loss prior to initial presentation were also recorded.

The lung cancer survey classified morphology into either small cell lung cancer (SCLC), non-small cell lung cancer (NSCLC) or not pathologically confirmed (NPC). SCLC disease stage was classified according to the Veteran’s Administration staging system [12] and categorised into limited, extensive or unknown. For comparison with other data sets, limited stage was considered to be “localised” and extensive stage was considered to be “non-localised” disease. For cases with NSCLC or NPC, disease stage was recorded in terms of tumour stage, nodal involvement and distant metastases (TNM). Disease stage was defined as localised (tumour size T0-T2 and no known nodal involvement or metastases), non-localised (T3-T4 or nodal involvement or presence of metastases) or unknown. The colorectal cancer survey classified disease stage into localised (involvement of the submucosa or muscularis propria with no known nodal involvement or metastases), non-localised (subserosa or serosal involvement, adjacent organ invasion, nodal involvement or distant metastases) or unknown.

### Routinely collected health data

The administrative data sources have been described previously [2]. Briefly, the CCR is notified of all cancer diagnoses in NSW and collects information including month and year of diagnosis, cancer site and spread of disease at diagnosis. The latter was defined as localised, non-localised (adjacent organs or regional lymph nodes involved, or distant metastases) and unknown. The CCR does not record treatment information. CCR records for people diagnosed with colorectal or lung cancer in NSW from January 1999 to December 2002 were included in the linkage.

The APDC collates procedures and diagnosis information for all admitted patient episodes in NSW public and private hospitals. Procedures were coded using the Medicare Benefits Schedule-Extended classification of the International Classification of Diseases 10th revision, Australian Modification (ICD-10-AM). Diagnosis information was recorded as the primary diagnosis and additional diagnoses (additional diagnoses affecting treatment or length of stay) and coded to ICD-10-AM. Up to 31 procedure codes and 40 diagnosis codes could be recorded for each admission. APDC records from July 1998 to June 2003 were included in the linkage to ensure full coverage of admissions relevant to the primary treatment of each cancer.

### Treatment and comorbidities

For APDC records, ICD-10-AM codes corresponding to the procedures recorded in the surveys were identified by cancer specialists. Chemotherapy and radiotherapy were identified using procedure codes and supplemented with diagnoses indicating that the treatment had been received (e.g. “Radiotherapy session”) or that the admission was related to convalescence or sequelae of the treatment (e.g. “Convalescence following chemotherapy”). Radiotherapy is not indicated for colon cancer patients so the evaluation of the recording of radiotherapy treatment was restricted to rectal and lung cancer cases.

Comorbidities were included in comparisons because of their important role in determining patterns of care. The presence of relevant comorbidities was identified among the APDC principal and additional diagnoses using the codes described by Quan et al. [13] for the Charlson Comorbidity Index [14]. We considered two different algorithms using records representing: (1) hospital episodes in the 12 months up to and including the month of diagnosis, plus the first cancer-related admission if it occurred after the month of diagnosis; and (2) all available hospital episodes for 1998–2003. Comparable comorbidities were ascertained only in the lung cancer survey, and as those ascertained did not correspond exactly to those in the Charlson Index, ischaemic heart disease was combined with other atherosclerotic disease recorded in the lung cancer survey to be compared with the combination of myocardial infarction, congestive heart failure and additional ischaemic heart disease diagnoses recorded in the APDC (referred to as “heart disease”).

### Record linkage

As described previously [2], the NSW Department of Health used probabilistic matching to link CCR and APDC records. The Centre for Health Record Linkage (CHeReL) then matched records from the CCR and APDC to those in the colorectal and lung cancer surveys using probabilistic matching [15]. Uncertain matches and a sample of “certain” matches were reviewed clerically. The CHeReL estimated that there were approximately 0.1% false positive and less than 0.1% false negative linkages.

The patterns of care studies and linkage processes were approved by the ethics committees of the NSW Department of Health, Cancer Institute NSW and Cancer Council NSW.

### Statistical analysis

Individual patient data provided by doctors or collected from doctors’ records in the colorectal and lung cancer surveys were compared with APDC records for diagnostic investigations and treatment (curative surgery, chemotherapy, or radiotherapy). For lung cancer patients only, they were also compared with disease stage data in the CCR (we have previously reported this comparison for colorectal cancer [7]), and with selected comorbid conditions in the APDC. All comparisons of survey observations with APDC records include only those patients who linked to at least one APDC record. For the purpose of this analysis, the survey data were considered to be the “gold standard”. Sensitivity was defined as the probability of an event being recorded in the administrative data if it was in the survey data and specificity was the probability of an event not being recorded in the administrative data if it was not in the survey data.

The local government area of the patient’s place of residence at the time of cancer diagnosis was used to determine their accessibility to services as defined by the Accessibility/Remoteness Index for Australia [16].

Using chi-square tests, the proportions of patients on which there was agreement between the surveys and administrative data were compared across groups defined by age, sex, remoteness of residence, year of diagnosis, and disease stage for all cases, and tumour morphology (recorded by the survey), performance status, weight loss and comorbidities for lung cancer cases. Analyses were carried out in SAS version 9.1 (SAS Institute Inc., Cary, NC, US).

## Results

**Figure 1:**
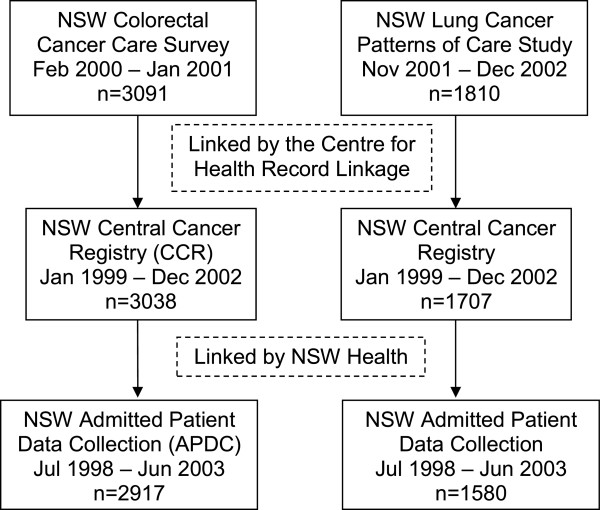
Data sources and linkage.

There were 3091 colorectal cancer cases and 1810 lung cancer cases with treatment data from the surveys. 3038 (98%) colorectal cancer cases and 1707 (94%) lung cancer cases successfully linked to the CCR (Figure 1). Of these, 2917 (96%) colorectal cancer cases and 1580 (93%) lung cancer cases linked to at least one APDC record and were included in the analyses (Figure 1, Table 1).

**Table 1:** Characteristics of patients in the NSW Colorectal Cancer Care Survey (n=2917) and the NSW Lung Cancer Patterns of Care Study (n=1580) who were linked to cancer registry and hospital records

	**Colorectal cancer survey**		**Lung cancer survey**	
	**n**	**%**	**n**	**%**
**Age (years)**				
<50	201	7	68	4
50-59	455	16	247	16
60-69	764	26	422	27
70-79	964	33	610	39
80+	533	18	233	15
**Sex**				
Female	1254	43	539	34
Male	1663	57	1041	66
**Cancer stage (TNM)**				
Localised	747	26	409	26
Non-localised	2106	72	1142	72
Unknown	64	2	29	2
**Remoteness of residence**^**a**^				
Highly accessible	2389	82	1239	78
Accessible	423	15	293	19
Moderately accessible	44	2	36	2
Remote/very remote	19	1	12	1
Unknown	42	1	0	0

^a^ Based on Accessibility/Remoteness Index for Australia (ARIA), using distance from the place of residence to major service centres.

TNM – classification using tumour stage, nodal involvement and distant metastases.

Colorectal cancer survey cases linked to the CCR and APDC were more likely than those who were not linked to be female (43% and 34% respectively, 95% confidence interval [CI] for difference: 1-16%), and to have had a colonoscopy (79% and 72%, 95% CI for difference: 0-14%). Lung cancer survey cases linked to the CCR and APDC were more likely than those who were not linked to have had surgery (17% and 8%, 95% CI for difference: 4-12%) and to have had a bronchoscopy (51% and 44%, 95% CI for difference: 0-14%). Failure to link to the CCR or APDC could have been due to insufficient matching of identifying details to be certain of a match. Not linking to the APDC could also have been due to not having any APDC inpatient hospital episodes (due to no hospital admission, non-recording of hospital episodes, or treatment outside NSW). Not linking to the CCR could have been due to the cancer not being registered in the CCR within the study period; early notification records were used to identify patients for the surveys.

### Disease stage

Non-localised lung cancer was the most common disease stage, accounting for 72% of cases in the survey and 57% of cases in the CCR. Eighteen percent of lung cancer cases had unknown disease stage in the CCR, compared to only 2% in the lung cancer survey. This contributed to the poor sensitivity for both non-localised (sensitivity 68%) and localised (sensitivity 52%) disease. There was agreement between the survey data and the CCR for 63% of cases. After excluding lung cancer cases with unknown disease stage in either source of information, there was agreement on stage for 77% of the 1283 cases and the specificity with which the CCR recorded non-localised disease was 65%.

### Diagnostic procedures

Only colonoscopies were recorded accurately in the APDC (Tables 2,3). The sensitivity with which the APDC recorded colonoscopies for colorectal cancer cases was highest for those treated in private hospitals (97%) or with private health insurance (97%) and lowest for those without health insurance (85%). Specificity was highest for cases aged 80 years or more (63%) and those treated in public hospitals (62%). There were 284 patients with a colonoscopy recorded in the APDC and not in the survey. Of these, 118 (42%) were after the month of diagnosis and were more likely to be related to post-treatment monitoring rather than pre-operative tests and thus might not have been captured in the survey.

**Table 2:** Diagnostic and treatment procedures recorded in the NSW Colorectal Cancer Care Survey and the APDC (n=2917)

	**Colorectal cancer survey**		**APDC**		**Sensitivity**^**a**^	**Specificity**^**a**^
	**n**	**%**	**n**	**%**	**%**	**%**
**Diagnostic investigations**	**2817**	**97**	**2592**	**89**	**89**	**23**
**Bowel visualisation**	**2612**	**90**	**2425**	**83**	**87**	**52**
Colonoscopy	2314	79	2396	82	91	53
Other^b^	768	26	142	5	9	97
**Imaging for distant metastases**	**2125**	**73**	**792**	**27**	**28**	**74**
Abdomino-pelvic CT scan	1501	51	708	24	28	80
Any abdomino-pelvic scan	1624	56	748	26	29	79
Any chest scan	1519	52	176	6	5	93
Bone scan	20	1	44	2	10	99
**Resection of primary cancer**	**2764**	**95**	**2654**	**91**	**96**	**92**
Colon resection	1393	48	1453	50	94	90
Rectal resection	1401	48	1240	43	85	96
Total proctocolectomy	16	1	45	2	63	99
**Other surgical resections**						
Liver resection	17	1	97	3	65	97
Oophorectomy	71	2	76	3	65	99
**Other treatment**						
Chemotherapy	1027	35	472	16	40	97
Radiotherapy for rectal cancer^c^	312	25	66	5	15	98

APDC: Admitted Patient Data Collection.

^a^ Sensitivity and specificity of the APDC compared with the colorectal cancer survey.

^b^ Includes barium enema, sigmoidoscopy and endorectal ultrasound.

^c^ 1226 rectal cancer cases.

**Table 3:** Diagnostic procedures, treatment procedures and comorbidities recorded in the NSW Lung Cancer Patterns of Care Study and the APDC (n=1580)

	**Lung cancer survey**		**APDC**		**Sensitivity**^**a**^	**Specificity**^**a**^
	**n**	**%**	**n**	**%**	**%**	**%**
**Diagnostic investigations**^**b**^	**1561**	**99**	**1045**	**66**	**66**	**47**
Chest CT scan	1430	91	430	27	27	74
Chest x-ray	1405	89	0	0	0	100
Brain CT scan	461	29	283	18	34	90
Bone scan	495	31	182	12	26	95
Bronchoscopy	805	51	563	36	65	95
Biopsy	664	42	671	42	64	73
**Resection of primary cancer**	**262**	**17**	**256**	**16**	**92**	**99**
Pneumonectomy	45	3	39	2	78	100
Lobectomy	198	13	153	10	70	99
Other resection	25	2	74	5	76	96
**Other treatment**						
Chemotherapy	478	30	230	15	36	96
Radiotherapy	626	40	222	14	30	96
**Comorbidities**						
COPD	599	38	295	19	35	91
Diabetes	164	10	152	10	74	98
Heart disease	363	23	137	9	25	96
One or more of the above	836	53	472	30	45	87

APDC: Admitted Patient Data Collection.

COPD: Chronic obstructive pulmonary disease.

^a^ Sensitivity and specificity of the APDC compared with the lung cancer survey.

^b^ Includes chest CT, chest x-ray, brain CT, bone scan, bronchoscopy, and biopsy.

The APDC recorded around two-thirds of the bronchoscopies and biopsies, but one-third or fewer of the radiography procedures for the diagnosis of lung cancer.

### Potentially curative surgical treatment

The sensitivity with which the APDC recorded potentially curative surgical treatment was over 90%, but the recording of the actual surgical procedure was less accurate (Tables 2,3). One-fifth of lobectomies for lung cancer were recorded as pneumonectomies or other definitive resections in the APDC. Four percent of rectal cancer cases who had a rectal resection recorded in the survey were recorded as having a colon resection in the APDC; the converse error occurred in 1% of colon cancer cases who had a colectomy.

The sensitivity with which any curative colorectal cancer surgical treatment was recorded in the APDC was lowest for cases with unknown disease stage (88%), but it was at least 94% for all other patient groups. Of the 123 cases in the colorectal cancer survey who had curative surgical treatment but no corresponding record in the APDC, 45 (37%) had a matching admission date in the APDC, with around a third of these having an intestinal resection or other (minor) rectal resection recorded.

The sensitivity with which any lung cancer surgical treatment was recorded in the APDC was lowest for cases from rural areas (78%, 95% in non-rural areas); there was no appreciable variation for any other patient groups. Excluding seven cases who were likely to have been treated interstate, all of whom were from rural areas, increased the sensitivity with which the APDC captured surgical treatment for cases from rural areas to 94%. Of the thirteen other cases who had undergone surgery according to the lung cancer survey but had no record of surgery in the APDC, eight had a non-surgical admission recorded in the APDC on the same day that the surgery recorded in the survey was performed.

For the cases who had surgery recorded in both sources, date of surgery differed slightly between the survey and administrative data for 20% of colorectal cancer cases and 16% of lung cancer cases with the majority having surgery up to a week earlier according to the APDC.

### Chemotherapy

The receipt of chemotherapy was under-enumerated in the APDC for both colorectal and lung cancer cases, with records in the APDC for less than half of the cases treated with chemotherapy according to the surveys (Tables 2,3). Of the cases identified in the APDC as having had chemotherapy, over 90% were identified from the procedure codes and the remainder were identified through relevant diagnosis codes only.

### Radiotherapy

Enumeration of radiotherapy treatment in the APDC was even lower than that for chemotherapy, with less than one-sixth of rectal and one-third of lung cancer cases treated with radiotherapy identified (Tables 2,3). Radiotherapy treatment recorded in the APDC was identified from diagnosis codes only for 80% of rectal cancer cases and one-third of lung cancer cases. The majority of diagnosis codes in the APDC that identified radiotherapy treatment indicated after-effects of treatment not radiotherapy administered during the hospital stay. There were five lung cancer cases who, according to the lung cancer survey, had radiotherapy after the end of the period covered by the APDC. These were the only survey treatment records outside the period covered by the APDC, and they account for only 1% of the 440 cases with lung cancer who had radiotherapy that was not captured in the APDC.

### Comorbidities

For key comorbidities, the level of agreement between the survey data and APDC for lung cancer cases was reasonable for diabetes but poor for COPD and heart disease (Table 3). When we considered comorbidities recorded in the APDC over the entire study period (our secondary analysis), the sensitivity with which each condition was recorded increased by 14-16% with only small reductions in specificity (e.g. 88% sensitivity and 96% specificity for the recording of diabetes).

## Discussion

Linked routinely collected administrative health data provided reasonably accurate information about curative surgery for colorectal and lung cancer cases, colonoscopies for colorectal cancer patients and comorbidities such as diabetes. The recording of disease stage was less accurate and the administrative data did not capture the majority of diagnostic investigations other than colonoscopies, nor comorbidities other than diabetes, nor treatment with chemotherapy or radiotherapy.

While surgical treatment was well enumerated overall, there were some discrepancies in the recording of specific surgical procedures. Other studies have reported that agreement was lower for less definitive and less commonly performed procedures, and this may relate to the interpretation of the surgeons’ notes [17,18]. We previously found that for prostate cancer, radical prostatectomy was recorded in the administrative data with 91% sensitivity and 100% specificity [2]. Another NSW study reported some mis-coding of mastectomies and breast conserving surgery [3]. Surgical treatment was not as well enumerated for cancer cases living in more rural areas, mainly due to data not being available for treatment in hospitals in neighbouring states.

Chemotherapy, radiotherapy and diagnostic investigations other than colonoscopy are often carried out on an outpatient basis, so analyses using inpatient episodes only are expected to under-enumerate the use of these procedures. Radiotherapy appeared more likely to be identified for either cancer type when a long hospital admission coincided with the patient having radiotherapy. In contrast, previous research found that radiotherapy in the form of brachytherapy for prostate cancer patients was enumerated accurately as it requires a specific hospital admission [2].

Our results concur with previous studies using NSW linked administrative health data that reported a small under-enumeration of cancer-specific surgery [2,3] and a larger shortfall for radiotherapy [2]. Other Australian and international validation studies have also reported high accuracy for major surgical procedures [18-21] in administrative data collections, reasonable recording of disease stage [7-9] and under-enumeration of diagnostic investigations, chemotherapy and radiotherapy [20-23]. We previously found that the inclusion of Australian Medicare claims data substantially improved the enumeration of radiotherapy and also captured many of the cases receiving surgery who were missed by the APDC [2].

While the presence of diabetes was reasonably well captured for lung cancer cases, the other comorbidities investigated were vastly under-enumerated. Others have also reported that routinely collected diagnosis information under-enumerates comorbidities with the possible exception of diabetes [24-26]. This may be due to the comorbidity information being collected in the administrative data and surveys for different purposes. It may also depend on the period over which comorbidity is enumerated, as we found that sensitivity of APDC recording of comorbidity increased when we enumerated it over a longer period. While it seems that hospital records do under-report information on comorbidities, the available comorbidity data are still important when assessing patient outcomes [25,27].

The poor agreement between the administrative and survey data with regards to cancer stage suggests that we cannot judge the appropriateness of treatment based solely on administrative data [7,28]. The colorectal and lung cancer surveys recorded detailed information on tumour stage, lymph node involvement, site(s) of distant spread, patient performance status, weight loss prior to presentation, patient preferences (with respect to choice of treatment) and quality of life, thus providing a more comprehensive picture of cancer management.

We excluded cancer cases who did not link to the APDC so our estimates of sensitivity for procedures are likely to be somewhat optimistic. When all cases who did not link to the APDC were considered not to have had any of the procedures according to the administrative data, the sensitivity was reduced by 3-5% for each of the major procedure types and comorbidities.

Our study has other limitations. The comorbid conditions that are recorded in the hospital data are those that caused the admission or had some effect on the hospital stay, so this might not capture all relevant comorbid conditions. Also, data we used might now be considered relatively old. However, we believe there have not been any major changes in data quality or treatment that would substantially alter the quality of more recent data, thus our results are still relevant.

The administrative data have some key strengths. First, they are population-based, which removes some of the potential biases introduced by single-centre data collections or other area-based samples. Second, perhaps most important in a research environment with finite funding and resources, the data are relatively inexpensive and timely to acquire and are already being collected by experts in the field, making it possible to undertake regular large-scale analyses.

How can the administrative data be used to provide more comprehensive information on cancer treatment patterns? Marginal gains are possible with improved quality and availability of patient identifiers (name, date of birth, etc.) for record linkage. However the under-enumeration of diagnostic procedures, chemotherapy and radiotherapy deserves more attention. The addition of other routinely collected data sources would help address this issue, in particular Medicare claims data, which have been shown to improve the accuracy of treatment and comorbidity information [2,24,29-31]. The use of treatment data recorded by clinical cancer registries in NSW would also be a step forward; although currently these registries do not cover all cancers diagnosed in NSW [32]. There is also a need for information that is not currently routinely recorded, such as performance status on admission and clinicians’ recommendations or patients’ preferences for treatment. These data may only be possible through patient or clinician surveys, although well designed and well functioning electronic medical record systems could facilitate their collection.

## Conclusions

Overall, the linked routinely collected administrative health data we used accurately described the overall use of potentially curative surgery for colorectal and lung cancer patients in NSW. This, combined with our previous findings for the treatment of prostate cancer, suggests that population cancer registries together with hospital admissions data are sufficiently accurate to monitor patterns of surgical care for different cancer types. Diagnostic procedures, chemotherapy, radiotherapy, comorbidities and cancer stage at diagnosis however, were not as well recorded in the administrative data, but information on colonoscopies might be sufficiently reliable. Information from other sources, such as Medicare claims data, is also required before routinely collected administrative data can be used to monitor cancer care at the population level.

## Abbreviations

APDC: Admitted Patient Data Collection; CCR: Central Cancer Registry; CHeReL: Centre for Health Record Linkage; ICD-10-AM: International Classification of Diseases 10th revision, Australian Modification; NPC: Not pathologically confirmed; NSCLC: Non-small cell lung cancer; NSW: New South Wales; SCLC: Small cell lung cancer; TNM: Tumour stage, nodal involvement and distant metastases.

## Competing interests

The authors declare that they have no competing interests.

## Authors’ contributions

DG performed the statistical analysis and drafted the manuscript. KA, LS and BKA guided the analysis and helped to draft the manuscript. DLO designed the study, guided the analysis and helped to draft the manuscript. All authors read and approved the final manuscript.

## Pre-publication history

The pre-publication history for this paper can be accessed here:

http://www.biomedcentral.com/1472-6963/12/387/prepub

## References

[B1] Tracey E Kerr T Dobrovic A Cancer in NSW: incidence and mortality report 2008 2010 Cancer Institute NSW, Sydney, Australia

[B2] Goldsbury DE Smith DP Armstrong BK O'Connell DL Using linked routinely collected health data to describe prostate cancer treatment in New South Wales, Australia: a validation study BMC Health Serv Res 2011 11 253 10.1186/1472-6963-11-253 21978077 PMC3206422

[B3] McGeechan K Kricker A Armstrong B Stubbs J Evaluation of linked cancer registry and hospital records of breast cancer Aust N Z J Public Health 1998 22 765 770 10.1111/j.1467-842X.1998.tb01490.x 9889440

[B4] Hayen A Smith DP Patel MI O'Connell DL Patterns of surgical care for prostate cancer in NSW, 1993–2002: rural/urban and socio-economic variation Aust N Z J Public Health 2008 32 417 420 10.1111/j.1753-6405.2008.00272.x 18959543

[B5] Thompson B Baade P Coory M Carriere P Fritschi L Patterns of surgical treatment for women diagnosed with early breast cancer in Queensland Ann Surg Oncol 2008 15 443 451 10.1245/s10434-007-9584-4 17909915

[B6] Hall SE Holman CD Platell C Sheiner H Threlfall T Semmens J Colorectal cancer surgical care and survival: do private health insurance, socioeconomic and locational status make a difference? ANZ J Surg 2005 75 929 935 10.1111/j.1445-2197.2005.03583.x 16336380

[B7] Yu XQ O'Connell DL Gibberd RW Abrahamowicz M Armstrong BK Misclassification of colorectal cancer stage and area variation in survival Int J Cancer 2008 122 398 402 10.1002/ijc.23043 17724717

[B8] Cunningham R Sarfati D Hill S Kenwright D An audit of colon cancer data on the New Zealand cancer registry N Z Med J 2008 121 46 56 18709047

[B9] Krnjacki LJ Baade PD Lynch BM Aitken JF Reliability of collecting colorectal cancer stage information from pathology reports and general practitioners in Queensland Aust N Z J Public Health 2008 32 378 382 10.1111/j.1753-6405.2008.00259.x 18782404

[B10] Armstrong K O'Connell DL Leong D Spigelman AD Armstrong BK The New South Wales Colorectal Cancer Care Survey - part 1: surgical management 2004 Cancer Council NSW, Sydney, Australia

[B11] Vinod SK O'Connell DL Simonella L Delaney GP Boyer M Peters M Miller D Supramaniam R McCawley L Armstrong B Gaps in optimal care for lung cancer J Thorac Oncol 2008 3 871 879 10.1097/JTO.0b013e31818020c3 18670305

[B12] Micke P Faldum A Metz T Beeh KM Bittinger F Hengstler JG Buhl R Staging small cell lung cancer: veterans administration lung study group versus international association for the study of lung cancer–what limits limited disease? Lung Cancer 2002 37 271 276 10.1016/S0169-5002(02)00072-7 12234695

[B13] Quan H Sundararajan V Halfon P Fong A Burnand B Luthi JC Saunders LD Beck CA Feasby TE Ghali WA Coding algorithms for defining comorbidities in ICD-9-CM and ICD-10 administrative data Med Care 2005 43 1130 1139 10.1097/01.mlr.0000182534.19832.83 16224307

[B14] Charlson ME Pompei P Ales KL MacKenzie CR A new method of classifying prognostic comorbidity in longitudinal studies: development and validation J Chronic Dis 1987 40 373 383 10.1016/0021-9681(87)90171-8 3558716

[B15] Centre for Health Record Linkagehttp://www.cherel.org.au; cited 22 October 2012

[B16] Department of Health and Aged Care Measuring remoteness: accessibility/remoteness index of Australia (ARIA) Occasional paper New series No. 14 2001 Revised Edition Canberra, Australia

[B17] MacIntyre CR Ackland MJ Chandraraj EJ Pilla JE Accuracy of ICD-9-CM codes in hospital morbidity data, Victoria: implications for public health research Aust N Z J Public Health 1997 21 477 482 10.1111/j.1467-842X.1997.tb01738.x 9343891

[B18] Pinfold SP Goel V Sawka C Quality of hospital discharge and physician data for type of breast cancer surgery Med Care 2000 38 99 107 10.1097/00005650-200001000-00011 10630724

[B19] Roos LL Gupta S Soodeen RA Jebamani L Data quality in an information-rich environment: Canada as an example Can J Aging 2005 24 Suppl 1 153 170 16080132 10.1353/cja.2005.0055

[B20] Quan H Parsons GA Ghali WA Validity of procedure codes in international classification of diseases, 9th revision, clinical modification administrative data Med Care 2004 42 801 809 10.1097/01.mlr.0000132391.59713.0d 15258482

[B21] Feigl P Glaefke G Ford L Diehr P Chu J Studying patterns of cancer care: how useful is the medical record? Am J Public Health 1988 78 526 533 10.2105/AJPH.78.5.526 3354736 PMC1349332

[B22] Malin JL Kahn KL Adams J Kwan L Laouri M Ganz PA Validity of cancer registry data for measuring the quality of breast cancer care J Natl Cancer Inst 2002 94 835 844 10.1093/jnci/94.11.835 12048271

[B23] Bickell NA Chassin MR Determining the quality of breast cancer care: do tumor registries measure up? Ann Intern Med 2000 132 705 710 10787363 10.7326/0003-4819-132-9-200005020-00004

[B24] Klabunde CN Harlan LC Warren JL Data sources for measuring comorbidity: a comparison of hospital records and medicare claims for cancer patients Med Care 2006 44 921 928 10.1097/01.mlr.0000223480.52713.b9 17001263

[B25] Malenka DJ McLerran D Roos N Fisher ES Wennberg JE Using administrative data to describe casemix: a comparison with the medical record J Clin Epidemiol 1994 47 1027 1032 10.1016/0895-4356(94)90118-X 7730905

[B26] Green J Wintfeld N How accurate are hospital discharge data for evaluating effectiveness of care? Med Care 1993 31 719 731 10.1097/00005650-199308000-00005 8336511

[B27] Humphries KH Rankin JM Carere RG Buller CE Kiely FM Spinelli JJ Co-morbidity data in outcomes research: are clinical data derived from administrative databases a reliable alternative to chart review? J Clin Epidemiol 2000 53 343 349 10.1016/S0895-4356(99)00188-2 10785564

[B28] Schifano P Papini P Agabiti N Scarinci M Borgia P Perucci CA Indicators of breast cancer severity and appropriateness of surgery based on hospital administrative data in the Lazio Region, Italy BMC Public Health 2006 6 25 10.1186/1471-2458-6-25 16464258 PMC1420286

[B29] Cooper GS Yuan Z Stange KC Dennis LK Amini SB Rimm AA Agreement of Medicare claims and tumor registry data for assessment of cancer-related treatment Med Care 2000 38 411 421 10.1097/00005650-200004000-00008 10752973

[B30] Du X Freeman JL Goodwin JS Information on radiation treatment in patients with breast cancer: the advantages of the linked Medicare and SEER data. Surveillance, epidemiology and End results J Clin Epidemiol 1999 52 463 470 10.1016/S0895-4356(99)00011-6 10360342

[B31] Warren JL Harlan LC Fahey A Virnig BA Freeman JL Klabunde CN Cooper GS Knopf KB Utility of the SEER-Medicare data to identify chemotherapy use Med Care 2002 40 Suppl 8 IV-55 IV-61 10.1097/01.MLR.0000020944.17670.D7 12187169

[B32] NSW Cancer Institute NSW clinical Cancer Registryhttp://www.cancerinstitute.org.au/data-and-statistics/cancer-registries/nsw-clinical-cancer-registrycited 22 October 2012

